# Comprehensive Bioinformatics Analysis Reveals the Role of Shared Cuproptosis‐ and Ferroptosis‐Related DEG DLD in Abdominal Aortic Aneurysm

**DOI:** 10.1111/jcmm.70399

**Published:** 2025-02-06

**Authors:** Xingwei Hu, Lu Hu, Xiaoyun Si, Qian Feng, Yi Ma, Zhijiang Liu, Xiang He, Bei Shi

**Affiliations:** ^1^ Department of Cardiology Affiliated Hospital of Zunyi Medical University Zunyi China; ^2^ Department of Cardiology The First People's Hospital of Chenzhou Chenzhou Hunan China; ^3^ Department of Cardiology The Affiliated Hospital of Guizhou Medical University Guiyang Guizhou Province China; ^4^ Department of Geriatrics Pingxiang People's Hospital Pingxiang Jiangxi China; ^5^ Department of Cardiology, State Key Laboratory of Organ Failure Research, Nanfang Hospital Southern Medical University Guangzhou China

**Keywords:** abdominal aortic aneurysm, cuproptosis, DLD, ferroptosis

## Abstract

Ferroptosis plays a crucial role in the progression of abdominal aortic aneurysm (AAA). Cuproptosis, as a new mode of death, has some similarities with ferroptosis. The primary objective of this study was to develop the role of shared cuproptosis‐related differentially expressed genes (CRDEGs) and ferroptosis‐related differentially expressed genes (FRDEGs) in AAA. RNA sequencing and bioinformatic analyses of human AAA tissue were used to identify dihydrolipoamide dehydrogenase (DLD), which is involved in cuproptosis and ferroptosis. qRT‐PCR and IHC assays further confirmed that the DLD level was substantially higher in the AAA group than in the control group. Finally, experimental verification was conducted to identify that DLD could promote the necrosis, apoptosis and mitophagy of SMCs. In summary, our research identified DLD, linked to cuproptosis and ferroptosis, as differentially expressed in AAA across human and murine samples. DLD's role in regulating SMC necrosis, apoptosis and mitophagy positions it as a potential AAA biomarker and therapeutic target, warranting further investigation for clinical applications.

**Abbreviations**
AAA: abdominal aortic aneurysm
DEG: differentially expressed gene
DLD: dihydrolipoamide dehydrogenase
PPE: Porcine pancreas elastase
qRT‐PCR: quantitative real‐time PCR
SMC: smooth muscle cell

## 1 Introduction

Abdominal aortic aneurysm (AAA) is defined by a localised expansion of the aortic diameter by over 50% more than the surrounding normal aortic tissue, or by a dilation that exceeds 3 cm in comparison to the adjacent normal arterial segment [1]. According to epidemiological research, the occurrence of AAA ranges from 1.9% to 18.5% among men and from 0% to 4.2% among women [2]. Studies have shown that in most cases, AAAs measure less than 5.5 cm in diameter, which means that surgical treatment is not required for the majority of these patients [3]. However, there is a growing incidence of smaller AAAs (less than 5.5 cm), which underscores the pressing need for treatments that can prevent or slow their enlargement [4]. Although nonpharmacological interventions such as quitting smoking, controlling blood pressure, managing lipid levels and regulating blood sugar are standard approaches to slow AAA progression, effective pharmacological treatments that can stop or slow the growth of an aneurysm are still lacking [5]. Understanding the mechanisms behind the development and advancement of AAA is a promising route for developing preventative or delaying strategies. Consequently, the goal of our study is to enhance our comprehension of the aetiology of AAA.

Ferroptosis, a specific form of regulated cell death (RCD) characterised by its iron‐dependent nature and initiation through the accumulation of lipid peroxides on cellular membranes [6], plays a significant role in the progression and responsiveness to treatment in AAA. Over the past few years, emerging studies have suggested that inhibiting ferroptosis effectively suppresses AAA formation. Numerous ferroptosis‐related differentially expressed genes (FRDEGs), such as ganglioside GM3 [7] and HMGB2 [8], have been identified as intervention targets for inhibiting AAA development. Moreover, recent research reports indicate that ferroptosis‐related genes hold promise as potential biomarkers for AAA [9, 10]. These findings contribute to a deeper comprehension of AAA pathogenesis and offer valuable insights for guiding treatment strategies. Recent investigations have shed light on the role of cuproptosis in a variety of diseases. Cuproptosis stems from the direct interaction between copper ions and fatty acylated components within the tricarboxylic acid cycle of mitochondrial respiration [11]. This interaction triggers the aggregation of fatty acylated proteins, downregulation of iron–sulphur cluster proteins and eventual cell death. Prior studies have identified cuproptosis‐related differentially expressed genes (CRDEGs) as pivotal biomarkers for conditions such as myocardial infarction [12, 13] and Crohn's disease [14]. Furthermore, a recent bioinformatics analysis of cuproptosis in AAA found that CRDEGs (NLRP3, FDX1) might play a potential role in AAA.

The recognition of similarities between ferroptosis and cuproptosis, both triggered by reactive oxygen species (ROS), suggests shared mechanisms in their involvement in diseases [15, 16]. Exploring genes associated with both processes can provide a more comprehensive understanding, potentially aiding in disease treatment and prognosis prediction. Previous research has successfully utilised shared cuproptosis‐ with ferroptosis‐related genes to predict the prognosis of patients with hepatocellular carcinoma (HCC), showcasing their significant predictive value [17]. However, the relationship between the shared cuproptosis‐ with ferroptosis‐related DEGs and the AAA progression is also not fully understood. In addition, the function role of shared cuproptosis‐ and ferroptosis‐related DEGs in AAA has not been verified.

In the present investigation, we employed bioinformatics techniques and subsequent validation using AAA patient samples to ascertain that dihydrolipoamide dehydrogenase (DLD) is a gene that is potentially common to both CRDEGs and FRDEGs within the pathology of AAA. Our innovative findings revealed that DLD is highly expressed in AAA tissue samples. Additionally, our study demonstrated that DLD plays a regulatory role in the necrosis, apoptosis and mitophagy of SMCs. Consequently, the differentially expressed gene (DEG) DLD, which is implicated in both cuproptosis and ferroptosis, may serve as a diagnostic biomarker for AAA.

## 2 Materials and Methods

### 2.1 Data Sources

The utilisation of publicly available datasets from the Gene Expression Omnibus (GEO) (http://www.ncbi.nlm.nih.gov/geo/) for investigating the pathophysiology of AAA is a common and valuable approach in research. Since these datasets are publicly available and de‐identified, they are often considered exempt from the need for patient consent or ethical clearance. In the specific dataset GSE47472, which includes mRNA expression profiles, 14 samples derived from AAA tissues and 8 samples from healthy tissues were employed for analysis. Utilising data from such repositories not only saves time and resources but also allows researchers to integrate information from multiple studies, enhancing the depth and breadth of their investigations into the molecular underpinnings of diseases like AAA.

### 2.2 Patient Specimens

The procurement of samples from individuals who underwent AAA resection surgery at Zhongshan People's Hospital and NanFang Hospital followed established procedures [18]. Ethical guidelines and protocols for handling human specimens were strictly adhered to and received authorisation from NanFang Hospital [18]. Aneurysm tissues obtained from the surgeries underwent specific preservation methods based on the intended analyses. Some samples were preserved in formalin for subsequent immunohistochemical staining, allowing for the visualisation of specific proteins within the tissue. Meanwhile, other samples were promptly frozen fresh in liquid nitrogen, preserving the biological material for later western blotting, quantitative polymerase chain reaction (qPCR) analysis and additional immunohistochemistry studies. These meticulous procedures ensured the integrity of the samples for various molecular and histological analyses.

### 2.3 Experimental Animals

The animal experimentation procedures strictly adhered to the guidelines delineated by the US National Institutes of Health's Guide for the Care and Use of Laboratory Animals and received prior approval from the Animal Research Committee at Southern Medical University. In the CaCl_2_‐induced AAA model, male C57BL/6 mice aged 10–12 weeks were employed [19]. The abdominal aorta, situated below the renal arteries and bifurcation of the iliac arteries, was meticulously isolated from the surrounding retroperitoneal structures. Subsequently, the external surface of the aorta underwent treatment with a cotton gauze soaked in 0.5 mol/L CaCl_2_ for a duration of 15 min. As a sham control, a parallel procedure was conducted using NaCl (0.9%) instead of CaCl_2_. Following treatment, the aorta was thoroughly rinsed with 0.9% sterile saline, and the incision was meticulously sutured. After a stipulated period of 3 weeks, mice with CaCl_2_‐induced AAA were humanely euthanised, and the abdominal aortas were extracted for subsequent immunohistochemistry and immunofluorescence analyses, adhering to ethical and procedural standards in animal research.

### 2.4 Functional Enrichment Analysis of Candidate Genes

We utilised the Venn Diagram tool, hosted at http://bioinformatics.psb.ugent.be/webtools/Venn/, to identify the common genes between the microarray data analysis and the GeneCards database, resulting in a list of putative candidate genes. Next, we subjected the candidate genes to Gene Ontology (GO) and Kyoto Encyclopedia of Genes and Genomes (KEGG) enrichment analyses using the ‘ClusterProfiler’ package in R (http://www.bioconductor.org/packages/release/bioc/html/clusterProfiler.html) to determine the affected cellular processes and signalling pathways associated with the potential and key targets. To evaluate the findings, a significance threshold of *p* < 0.05 was utilised.

### 2.5 Immunohistochemistry Staining

The 5‐μm specimen slides (patient specimens and experimental animal specimens) underwent deparaffinisation, followed by the quenching of endogenous peroxidase activity for 10 min with 3% (vol./vol.) hydrogen peroxide in 10% PBS. To block nonspecific binding sites, 10% bovine serum in PBS was added to the slides and incubated at room temperature for 1 h. Primary antibodies against ɑ‐SMA and DLD were then added to the slides and incubated overnight at 4°C, followed by the addition of a biotinylated secondary antibody at 37°C for 1 h. The slides were then treated with horseradish peroxidase (HRP)‐labelled streptavidin solution at 37°C and stained with diaminobenzidine, with haematoxylin serving as a counterstain [20].

### 2.6 Western Blotting

The protein extraction process from Human Aortic Smooth Muscle Cells (HASMCs) involved the use of a Protein Extraction Kit containing protease inhibitor PMSF and a Protein Phosphatase Inhibitor. The concentration of the extracted proteins was quantified with a BCA Protein Assay Kit (Beyotime, P0010). Subsequently, proteins were denatured at 100°C for 10 min and separated by electrophoresis on a 10% SDS‐PAGE gel. The separated proteins were then transferred onto a nitrocellulose membrane. Following membrane transfer, a blocking step with 10% bovine serum for 1 h at room temperature was implemented. The membrane was subsequently incubated with primary antibodies overnight at 4°C. After three washes of 15 min each, the membrane underwent incubation with a secondary antibody for 1 h at room temperature. Following another three washes, protein bands were detected using enhanced chemiluminescence (ECL Advance; No. RPN2235, GE Healthcare Life Sciences) and recorded with a ChemiDoc imaging system (Bio‐Rad Laboratories). β‐actin served as a negative control.

### 2.7 DEG Screening

The investigation of DEGs in the context of AAA and control samples was conducted by leveraging the standardised profiles of the gene expression matrix and the pertinent platform annotation information. The analysis was conducted using the limma package within the R statistical programming environment. We applied stringent criteria to identify DEGs, requiring a |log fold change (FC)| threshold > 1 and a *p*.adj < 0.05. Following the DEG identification, their expression profiles were illustrated through the creation of a heatmap, a process that was enabled by utilising the heatmap package in R. And the heat map normalises the data: normalises the rows. Row clustering is performed using Euclidean distance. Additionally, a Venn diagram analysis was performed to reveal the intersections and unique characteristics of the DEGs discovered. These analytical steps collectively provide a thorough and statistically sound investigation into the molecular distinctions that define AAA, thereby deepening our understanding of the intricate genetic factors at play in this medical condition.

### 2.8 Cell Culture and Transfection

HASMCs, a type of cell derived from the human aorta, are primarily responsible for the contraction and relaxation of blood vessel walls, as well as maintaining the structure and function of blood vessels. These cells were purchased from Guangzhou Geneseed Biotech Co. Ltd. HASMCs are maintained in an environment with Dulbecco's Modified Eagle Medium (DMEM), supplemented with 10% foetal bovine serum (FBS). Subsequently, the HASMCs were transferred to a serum‐deprived medium and exposed to either a DLD‐targeting plasmid or a nontargeting control plasmid. The cells were then cultured under conditions of 37°C with 5% CO_2_ in a humidified incubator for a period of 48 h. Following this treatment, the HASMCs were employed for further in vitro experimentation.

### 2.9 Masson's Trichrome Staining

Paraffin‐embedded sections of aortic tissue (patient specimens and experimental animal specimens) were treated to remove paraffin using xylene and rehydrated through a graded ethanol series (100% twice, then 90%, 80%, 70%), culminating in a water rinse. Staining commenced with a 5‐min application of Weigert's haematoxylin iron, followed by differentiation in a hydrochloric acid–ethanol solution. Subsequently, the sections were incubated with ponceau acid fuchsin for 5 min. The staining process continued with a 5‐min application of phosphomolybdic acid, followed by a 5‐min incubation with aniline blue or light green stain, sourced from Leagene in Beijing, China. To evaluate the extent of fibrosis, the fibrotic areas within the tissue samples were measured relative to the total left ventricular area. The quantification of fibrosis was accomplished utilising the ImageJ software.

### 2.10 Protein–Protein Interaction

The protein–protein interactions (PPI) among the DEGs were investigated utilising the Search Tool for the Retrieval of Interacting Genes/Proteins (STRING) database accessible at https://cn.string‐db.org/. Subsequently, the acquired data were visualised using Cytoscape software to depict the PPI networks. In order to discern the most pivotal genes within these networks, the CytoHubba plugin was employed. This plugin utilises the maximum neighbourhood component method, ranking genes based on their node gene degrees.

### 2.11 Statistical Analysis

The results are presented as mean ± standard deviation (SD). Statistical analysis was performed using SPSS version 20.0 (SPSS Inc., Chicago, IL). Normality tests were performed on all continuous variables. If variance equality among different groups was confirmed, unpaired Student's *t*‐test was used to analyse statistical differences between two independent groups. If normality could not be determined, nonparametric tests were applied, such as the Mann–Whitney *U* test for two independent groups. A *p* value of less than 0.05 was considered statistically significant [18].

## 3 Results

### 3.1 Identification of DEGs in GSE47472

**FIGURE 1:**
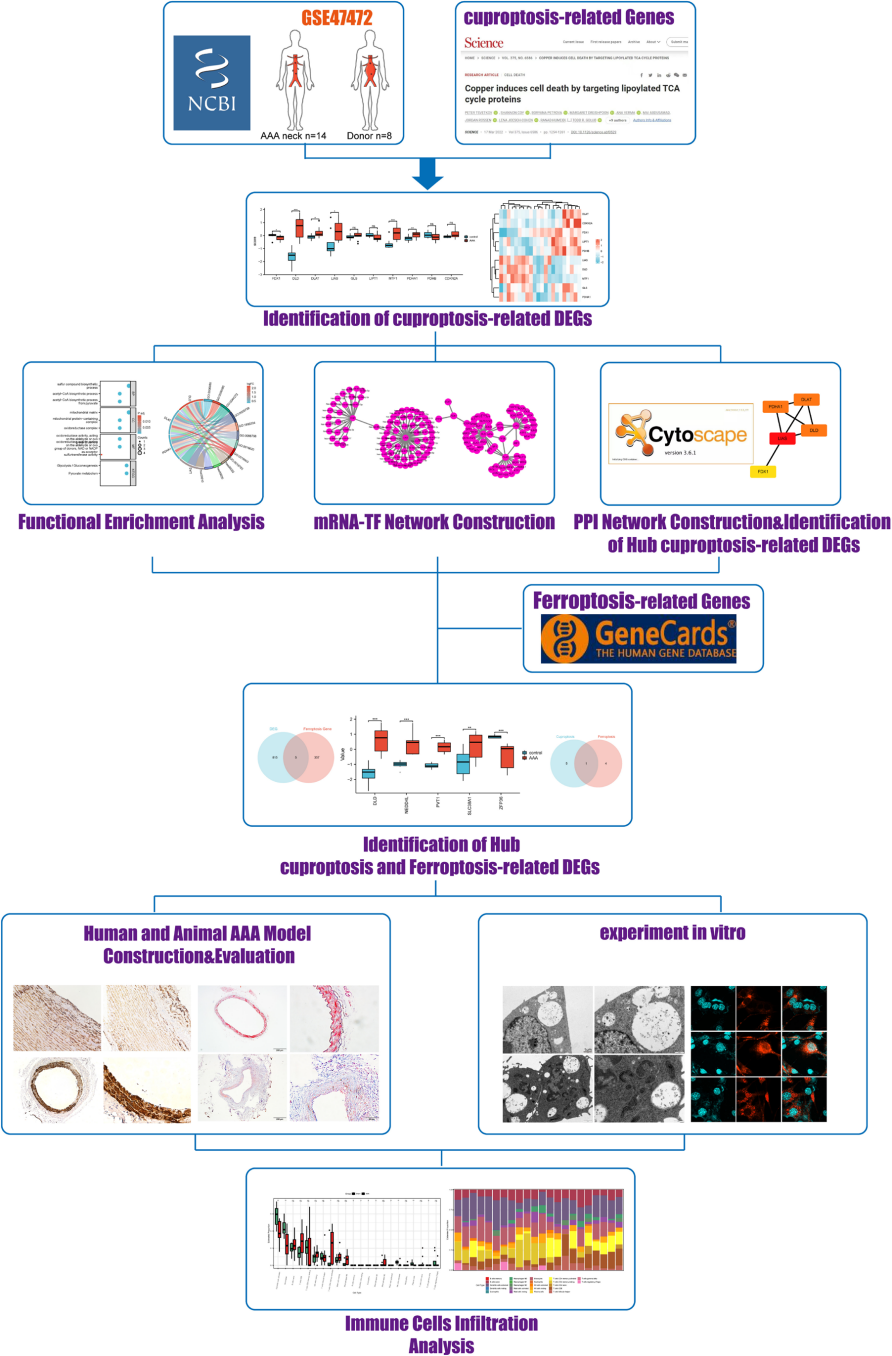
Workflow for the present study. AAA, abdominal aortic aneurysm; DEGs, differentially expressed genes; GO, Gene Ontology; KEGG, Kyoto Encyclopedia of Genes and Genomes; mRNA, messenger RNA.

**FIGURE 2:**
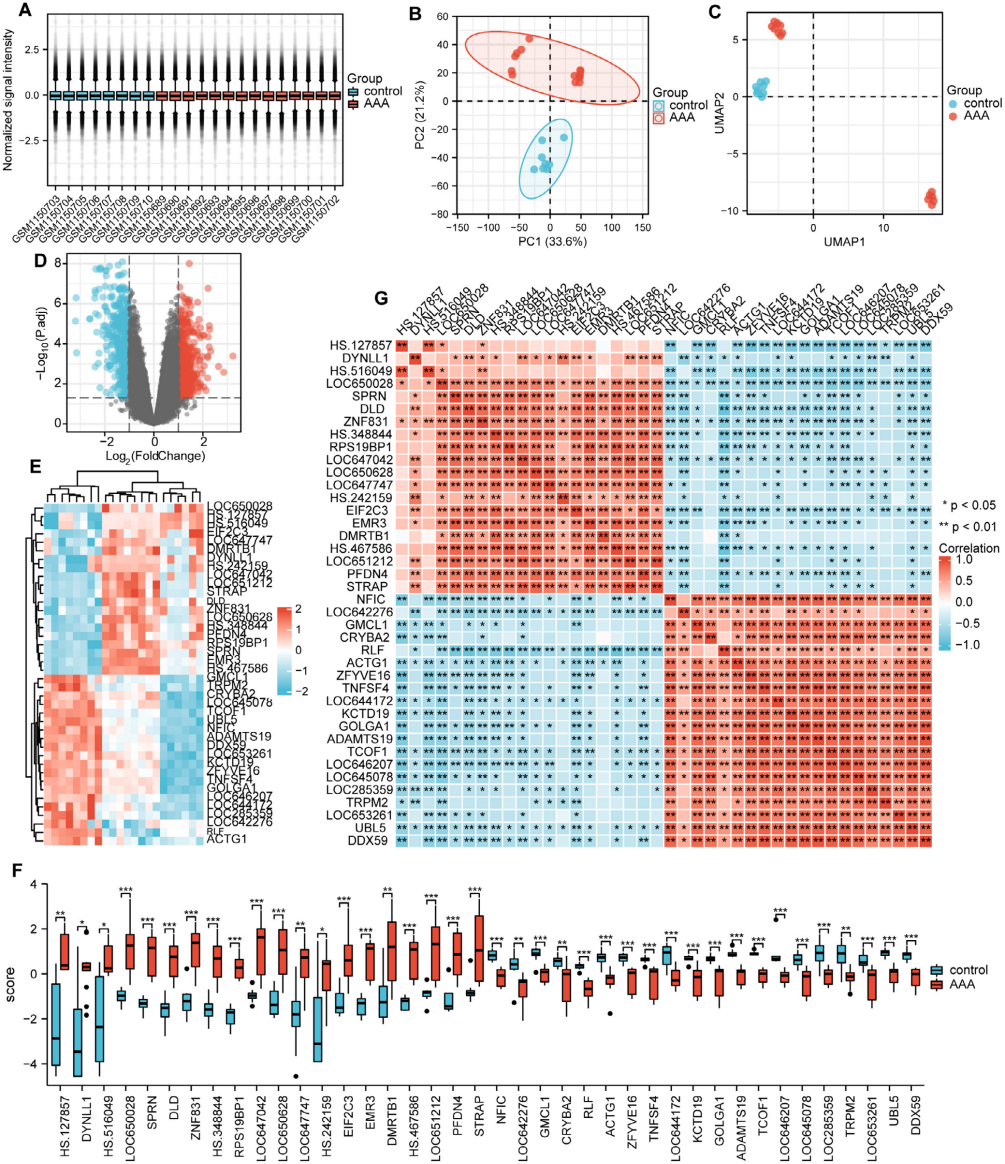
Identification of DEGs in GSE47472. (A) After data normalisation, a box plot was generated for each sample in the GSE47472 GEO datasets. (B, C) PCA plot and UMAP of the GSE47472 dataset. (D) The volcano plot shows the DEGs between control samples and AAA. The black points represent the adjusted *p* value > 0.05, blue points represent downregulated genes with an adjusted *p* value < 0.05, and red points represent upregulated genes with an adjusted *p* value < 0.05. (E, F) The expression levels and differences of the 20 upregulated and downregulated DEGs in GSE47472 dataset were visualised through a heat map and an expression difference map. (G) Correlation analysis was performed on the 20 upregulated and downregulated DEGs in GSE47472 dataset.

The workflow of the present study is presented in Figure 1. This passage describes the results of a study that investigated the gene expression profiles of patients with AAA compared to controls. The study analysed two datasets, GSE47472, using various methods including data normalisation, principal component analysis (PCA), uniform manifold approximation and projection (UMAP) and volcano plots to identify DEGs. The boxplot shows each sample after data normalisation (Figure 2A). The use of PCA and UMAP revealed distinct clusters of AAA samples and controls (Figure 2B,C). The volcano plots illustrated the aberrantly expressed RNA molecules (Figure 2D). We identified 2455 DEGs in the GSE47472 dataset, consisting of 389 upregulated and 431 downregulated genes. The study further analysed the top 20 upregulated and downregulated DEGs using a heatmap and group comparison chart (Figure 2E–G).

### 3.2 Identification of CRDEGs

**FIGURE 3:**
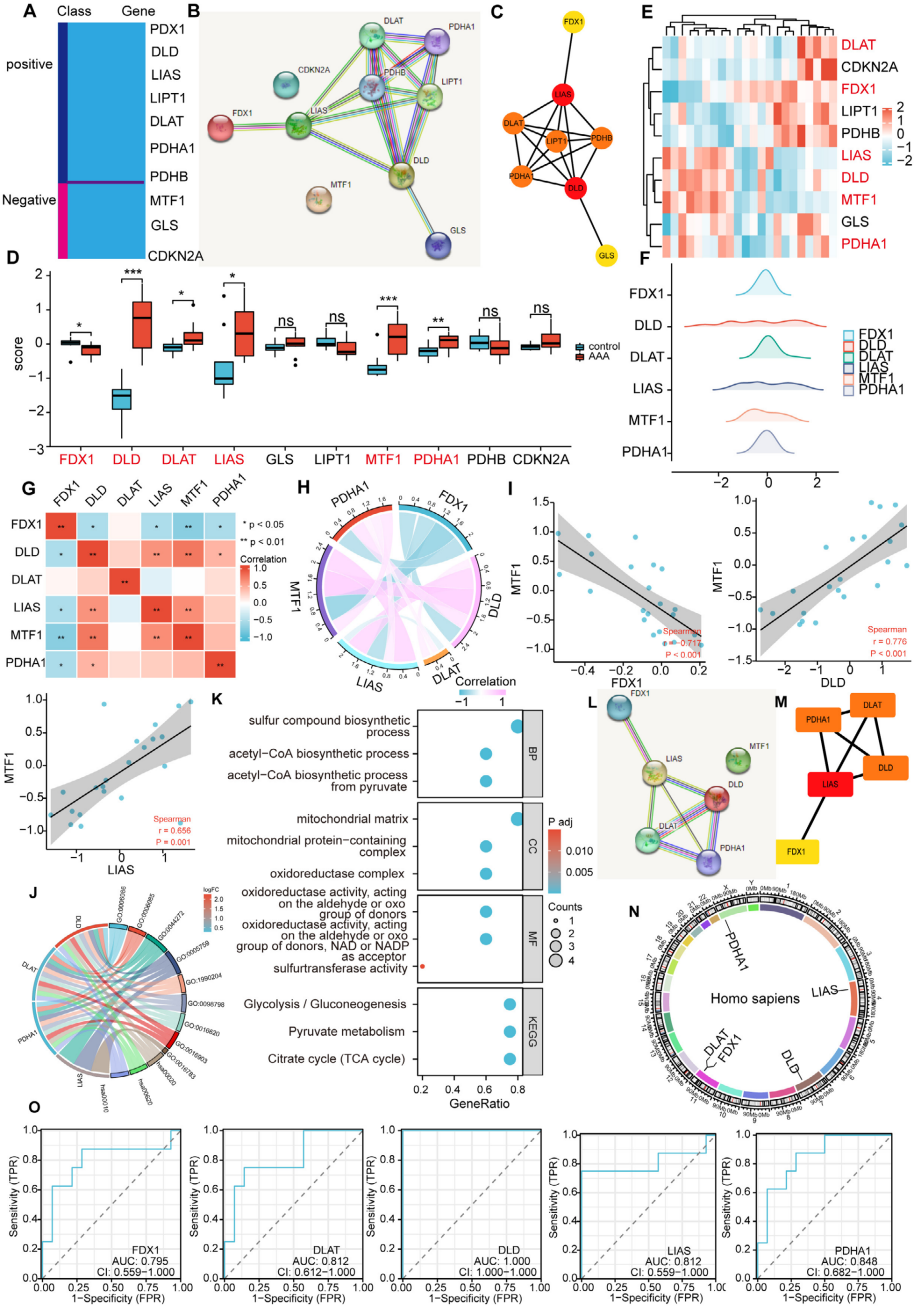
Identification of CRDEGs. (A) Classification of 10 cuproptosis‐related genes. (B. C) The STRING and Cytoscape software programs were used to create a PPI network of 10 cuproptosis‐related genes. (D, E) Expression difference map and heat map of the 10 cuproptosis‐related genes in GSE47472. (F) A mountain plot of six CRDEGs. (G–I) Correlation analysis of six CRDEGs in GSE47472. (J) A circular plot depicts the enriched Gene Ontology (GO) functional clusters of the six CRDEGs (K) The six CRDEGs related to AAA underwent Gene Ontology (GO) enrichment analysis in the categories of BP, CC, MF and KEGG. (L, M) PPI network of six CRDEGs. (N) Chromosomal map of five hub CRDEGs. (O) ROC curves were generated to evaluate the diagnostic value of five hub CRDEGs.

Cuproptosis‐related genes were identified based on information gathered from previous literature. The report indicated that FDX1, DLD, LIAS, LIPT1, DLAT, PDHA1 and PDHB exhibited a positive correlation with cuproptosis, while MTF1, GLS and CDKN2A displayed an opposite relationship (Figure 3A). To further explore the interactions among these cuproptosis‐related genes, a PPI network was constructed using the STRING database (Figure 3B). Subsequently, hub genes within this network were analysed using Cytoscape software, revealing that FDX1, DLD, LIAS, LIPT1, DLAT, GLS, PDHA1 and PDHB were identified as hub genes (Figure 3C). These hub genes may play pivotal roles in the regulation or manifestation of cuproptosis, and their comprehensive analysis could provide valuable insights into the underlying molecular mechanisms associated with this process. The results of differential analysis and heatmap of cuproptosis‐related genes suggested that FDX1, LIAS, DLD, DLAT, MTF1 and PDHA1 were differentially expressed. Compared with control group, LIAS, DLD, DLAT, MTF1 and PDHA1 were higher and FDX1 was lower in AAA (Figure 3D,E). Subsequently, a mountain plot was employed to visually represent the distribution of FDX1, LIAS, DLD, DLAT, MTF1 and PDHA1 within the dataset. The outcomes of this analysis revealed that the DLD gene exhibited a comparatively scattered distribution pattern in the GSE47472 dataset (Figure 3F). We investigated the correlation of FDX1, LIAS, DLD, DLAT, MTF1 and PDHA1 in the GSE47472 dataset. The results indicated that there was a correlation between them. As shown in the figure, red represents positive correlation, and blue represents negative correlation. The deeper the colour, the stronger the correlation. MTF1 and FDX1 exhibit a good negative correlation, while MTF1 and DLD or LIAS show a good positive correlation (Figure 3G,H). We will further analyse the correlation between MTF1 and FDX1, as well as between MTF1 and DLD or LIAS, using a correlation scatter plot (Spearman analysis). MTF1 is negatively correlated with FDX1 and is positively related to DLD and LIAS (Figure 3I). These results suggest that it plays an important role in the above relationship.

**FIGURE 4:**
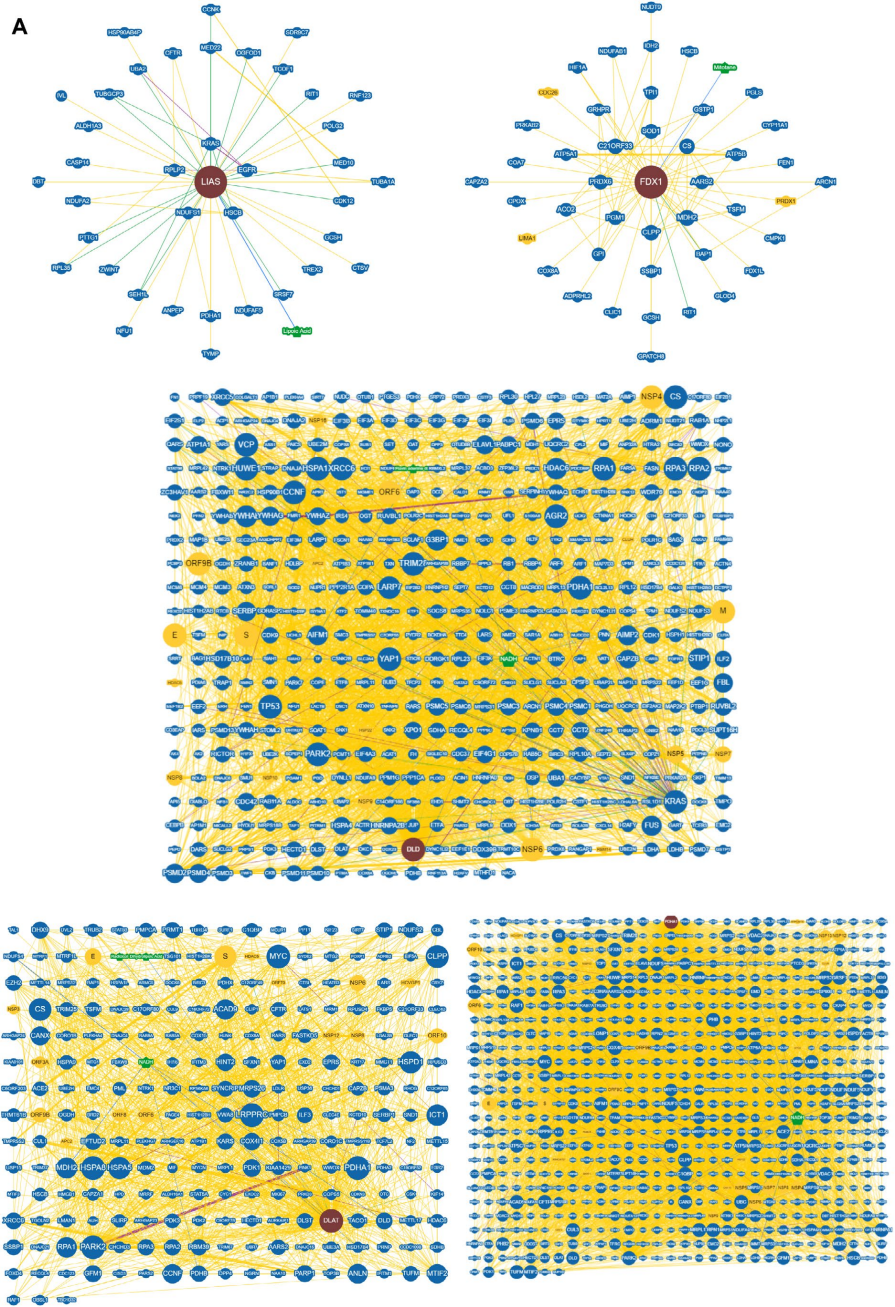
Prediction of five hub CRDEG interactions by BioGRID. (A) Prediction of the interacting proteins associated with FDX1, LIAS, DLD, DLAT and PDHA1 by BioGRID.

To explore the regulatory mechanism of differentially expressed cuproptosis‐related genes (FDX1, LIAS, DLD, DLAT, MTF1 and PDHA1), we executed GO and KEGG enrichment analyses using R software. In biological process (BP) analysis, these genes were mostly enriched in acetyl‐CoA biosynthetic process from pyruvate, acetyl‐CoA biosynthetic process and sulphur compound biosynthetic process. In cellular component (CC) analysis, these genes were mostly enriched in oxidoreductase complex, mitochondrial matrix and mitochondrial protein‐containing complex. In molecular function (MF) analysis, these genes were mostly enriched in oxidoreductase activity, acting on the aldehyde or oxo group of donors, NAD or NADP as acceptor, oxidoreductase activity, acting on the aldehyde or oxo group of donors and sulphur‐transferase activity. KEGG enrichment analyses showed that citrate cycle (TCA cycle), pyruvate metabolism and glycolysis/gluconeogenesis were mostly enriched (Figure 3J,K). To further screen cuproptosis hub genes, PPI network of differentially expressed cuproptosis‐related genes (FDX1, LIAS, DLD, DLAT, MTF1 and PDHA1) was constructed using the STRING database (Figure 3L). The top five hub genes were identified based on the CytoHubba Degree algorithm by Cytoscape software (Figure 3M). These included FDX1, LIAS, DLD, DLAT and PDHA1. To enhance the precision of the STRING database, we have conducted an in‐depth exploration of the interacting proteins associated with FDX1, LIAS, DLD, DLAT and PDHA1 by leveraging additional protein interaction databases, specifically BioGRID (Figure 4). The above results demonstrate that the top five hub genes—FDX1, LIAS, DLD, DLAT and PDHA1—have interconnections among themselves. For instance, PDHA1 is predicted to potentially interact with DLD, as well as with DLAT. Additionally, there are interactions between LIAS and PDHA1. Beyond these direct interactions, these genes are also associated with a wider network of proteins that play crucial roles in apoptosis, such as TP53. The results of chromosome localisation indicated that top five hub genes were located in the corresponding positions of chromosomes (Figure 3N). The receiver operating characteristic (ROC) curve analysis revealed that the signature of the top five hub genes possessed significant diagnostic utility. To determine the predictive efficacy of each individual gene, ROC curves were constructed for these genes. Figure 3O presents the ROC analysis outcomes for the top five hub genes, with each area under the curve (AUC) exceeding the threshold value of 0.5, indicating a high diagnostic accuracy.

### 3.3 The Five Hub CRDEGs and Their Interactions

**FIGURE 5:**
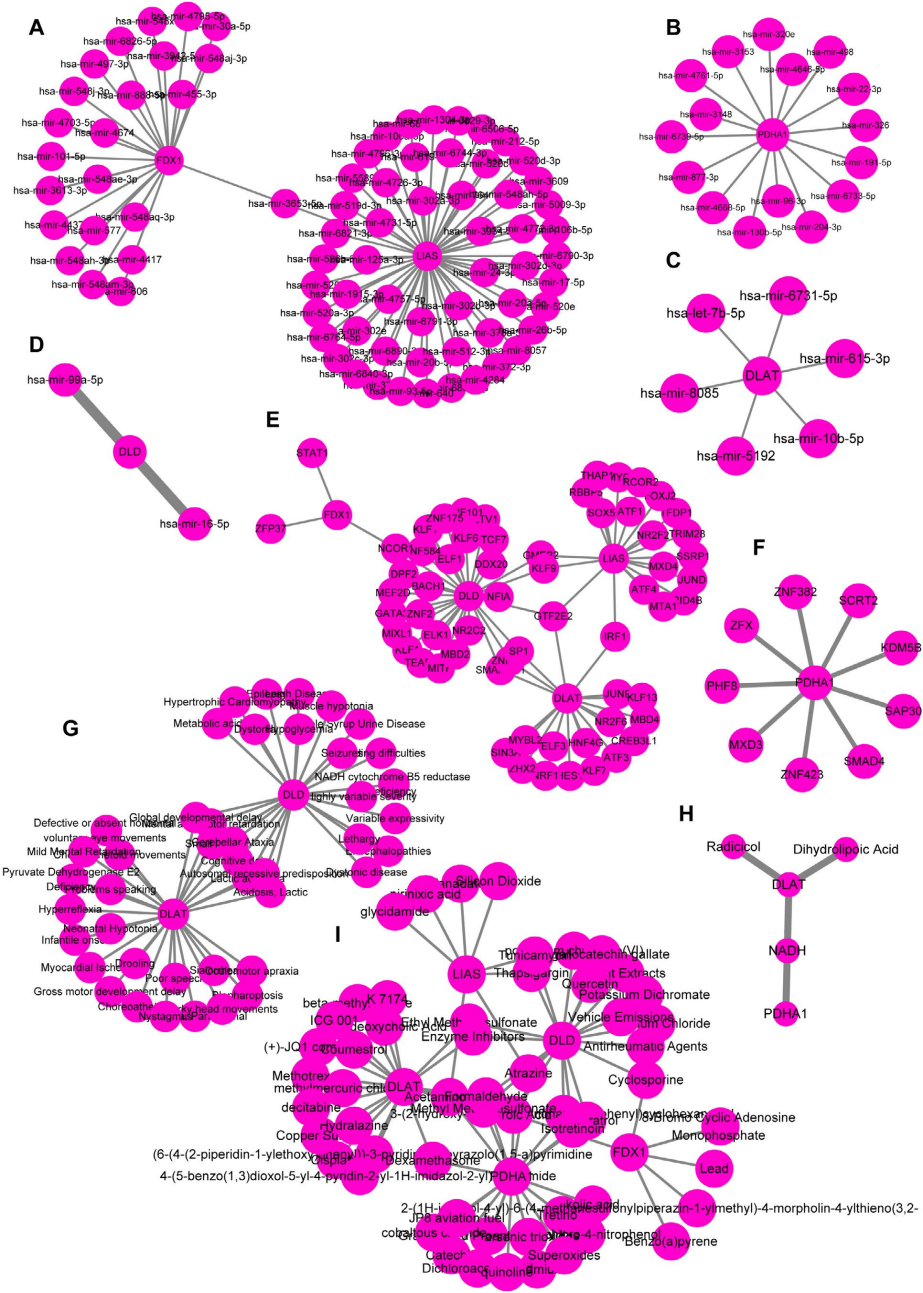
The five hub CRDEGs and their interactions. (A–D) The associated miRNA of five hub CRDEGs and their interactions. (E, F) The TF of five hub CRDEGs and their interactions. (G–I) The disease, drug and chemical interactions of five hub CRDEGs and their interactions.

The NetworkAnalyst was employed for the comprehensive analysis of miRNA, TF and drug interactions. The miRNA‐gene network, comprising five hub CRDEGs (FDX1, LIAS, DLD, DLAT and PDHA1) and their corresponding miRNAs, exhibited a complex network structure with a total of 106 nodes and 102 edges (Figure 5A–D). Within this intricate network, the five hub CRDEGs demonstrated close interactions through their targeting of specific miRNAs, such as let‐7b‐5p and miRNA‐24, both of which have been identified as differentially expressed in AAA. The TF‐gene network, encompassing the same five hub CRDEGs, displayed 76 nodes and 80 edges (Figure 5E,F). Furthermore, the analysis extended to hub CRDEGs' interactions with diseases, forming a network structure comprising 45 nodes and 51 edges (Figure 5G). Additionally, the investigation of hub CRDEGs–drugs interactions revealed potential therapeutic targets, exemplified by DLAT and PDHA1, capable of impeding AAA progression (Figure 5H). The protein–chemical interactions network, encompassing 5 hub CRDEGs and 56 chemicals, portrayed a comprehensive view with 61 nodes and 76 edges (Figure 5I). These integrated analyses provide a robust foundation for understanding the intricate regulatory networks involving miRNAs, TFs and drugs associated with CRDEGs in the context of AAA.

### 3.4 Identification and Verification of the Shared Cuproptosis and Ferroptosis‐Related DEG DLD in the AAA and Control Samples

**FIGURE 6:**
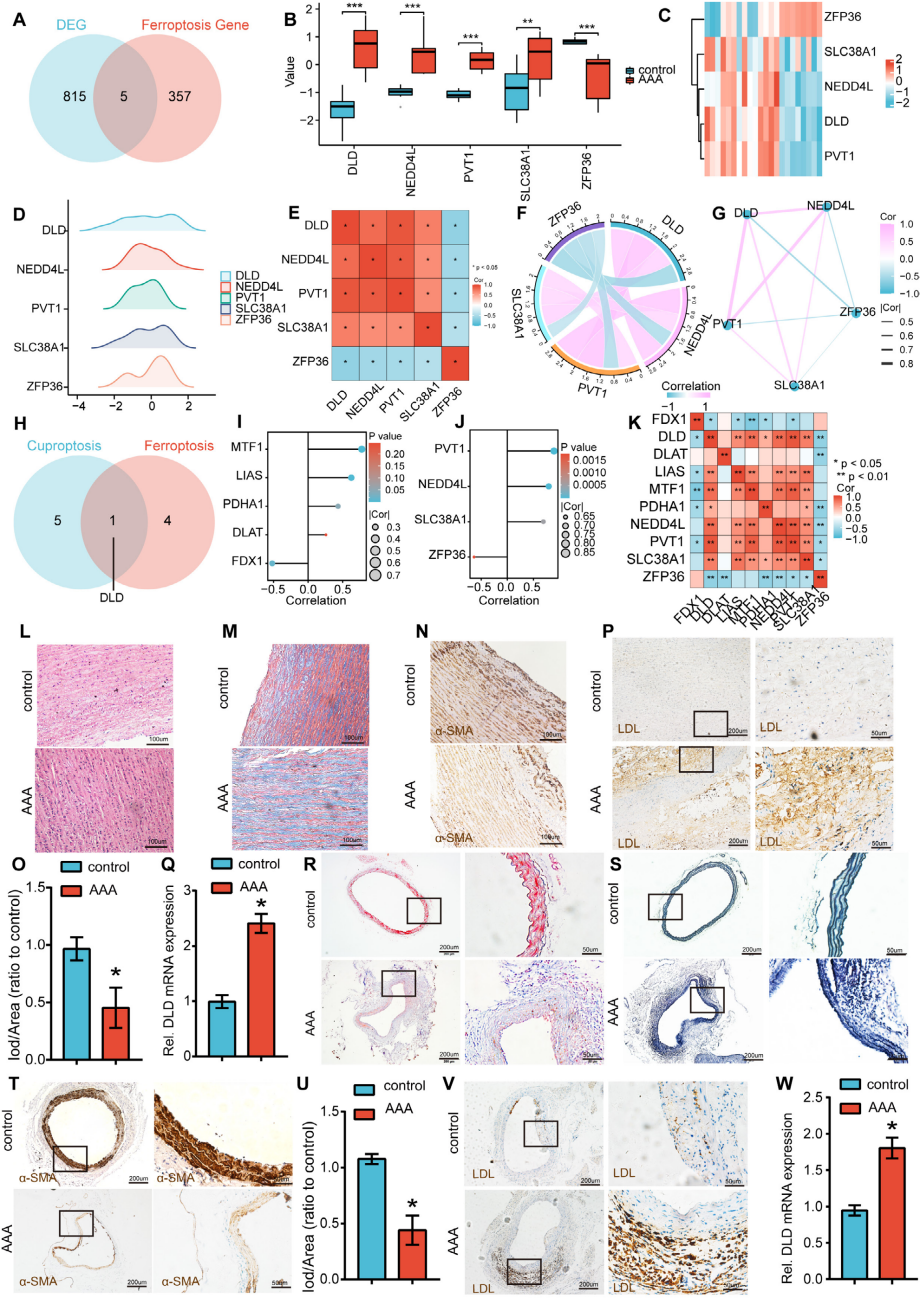
Identification and verification of the shared cuproptosis‐ and ferroptosis‐related DEG DLD in the AAA and control samples. (A) The Venn diagram illustrates the shared genes between the DEGs identified in the GSE47472 dataset and the ferroptosis‐related genes. (B,C) Expression difference map and heat map of the five FRDEGs in GSE7084. (D) A mountain plot of five FRDEGs. (E–G) Correlation analysis of five FRDEGs in GSE47472. (H) The Venn diagram illustrates the shared genes between six CRDEGs and the FRDEGs. (I) Correlation analysis between five CRDEGs and DLD in GSE47472. (J) Correlation analysis between four FRDEGs and DLD in GSE47472. (L) HE staining depicts the characteristics of AAA and adjacent normal aortic samples (bar = 100 μm). (M) Representative images of Masson trichrome‐stained artery sections of human AAA samples and normal aortic samples (bar = 100 μm). (N, O) Representative immunohistochemical staining and densitometric analysis of α‐SMA in human AAA samples and normal aortic samples (bar = 100 μm). (P) Representative immunohistochemical staining of DLD in human AAA samples and normal aortic samples (bar: Left 200 μm, right 50 μm). (Q) QRT‐PCR assay for detecting the expression level of DLD in human AAA samples and normal aortic samples. *p < 0.05 versus si‐scr; *n* = 5 per group. (R) Representative images of Masson trichrome‐stained artery sections of PPE‐induced AAA samples and normal aortic samples (bars: Left 200 μm, right 50 μm, magnified images). (S) Representative images of EVG stained artery sections of PPE‐induced AAA samples and normal aortic samples (bars: Left 200 μm, right 50 μm, magnified images). (T, U) Representative immunohistochemical staining and densitometric analysis of α‐SMA in PPE‐induced AAA samples and normal aortic samples (bars: Left 200 μm, right 50 μm, magnified images). (V) Representative immunohistochemical staining of DLD in AAA samples and normal aortic samples (bars: Left 200 μm, right 50 μm, magnified images). (W) QRT‐PCR assay for detecting the expression level of DLD in PPE‐induced AAA samples and normal aortic samples. *p < 0.05 versus si‐scr; *n* = 5 per group.

We overlapped ferroptosis‐related genes in GeneCard with the DEGs in GSE47472 and selected five shared FRDEGs (DLD, NEDD4L, PVT1, SLC38A1 and ZFP36) for further analyses. The Venn diagram analysis revealed five shared DEGs as FRDEGs (Figure 6A). The group comparison chart and expression heatmap of FRDEGs are presented. These results showed that FRDEGs DLD, NEDD4L, PVT1 and SLC38A1 were all highly expressed in AAA, while ZFP36 were low expressed in AAA (Figure 6B,C). A mountain plot was used to visually illustrate the distribution of DLD, NEDD4L, PVT1, SLC38A1 and ZFP36 within the dataset. The results of this analysis still indicated that the DLD gene maintained a relatively scattered distribution pattern in the GSE47472 dataset (Figure 6D). To assess the correlation among the five ferroptosis‐related DEGs (DLD, NEDD4L, PVT1, SLC38A1 and ZFP36) in the GSE47472 dataset, the results indicated that there was a correlation between them. ZFP36 is negatively correlated with DLD, NEDD4L, PVT1, SLC38A1 and DLD, while NEDD4L, PVT1 and SLC38A1 are positively correlated with each other (Figure 6E–G). To identify shared cuproptosis‐ and ferroptosis‐related DEG, we overlapped FRDEGs and CRDEGs and selected one shared cuproptosis‐ and ferroptosis‐related DEG DLD (Figure 6H). Our correlation analysis revealed that MTF1 had the highest level of association with DLD among the cuproptosis‐related genes (Figure 6I). Our correlation analysis revealed that PVT1 had the highest level of association with DLD among the ferroptosis‐related genes (Figure 6J). Furthermore, we performed a correlation analysis between cuproptosis‐related genes and ferroptosis‐related genes, revealing a correlation between them (Figure 6K). The results suggest that shared cuproptosis‐ and ferroptosis‐related DEG DLD is positively correlated with LIAS, MTF1, NEDD4L, PVT1 and SLC38A1, and negatively correlated with ZFP36 and FDX1.

Human AAA and corresponding adjacent normal aortic samples were collected from 10 patients who underwent AAA resection surgery. HE staining of aortic aneurysm and adjacent normal tissues revealed a discernible architectural disparity in SMCs. Specifically, SMCs within aneurysm samples displayed a disorganised arrangement, contrasting with the well‐ordered and parallel configuration observed in adjacent normal tissues (Figure 6L). Masson's trichrome staining further demonstrated a notable reduction in collagen deposition within the aortic walls of individuals diagnosed with AAA (Figure 6M). IHC staining results revealed that the expression of the SMC contractile marker α‐SMA was substantially decreased in AAA tissues compared with corresponding adjacent normal aortic tissues (Figure 6N,O). IHC staining confirmed that the levels of DLD were upregulated in the AAA samples compared with that in the control samples (Figure 6P,Q). We further investigated the expression of DLD in the AAA mouse model. The Masson's trichrome staining displayed diminished collagen deposition within the aortic tissue of the mouse model with AAA (Figure 6R). Additionally, the EVG staining showcased fragmented or disrupted elastic fibres specifically within the mouse AAA aorta (Figure 6S). Moreover, the parallel trends observed in the expression profiles of α‐SMA and DLD between human AAA and mouse AAA (Figure 6T–W) are noteworthy. These consistent trends in α‐SMA and DLD expression suggest a potential concordance in molecular signatures between human and mouse AAA.

### 3.5 DLD Regulates the Necrosis, Apoptosis and Mitophagy of SMCs and Macrophage Pyroptosis

**FIGURE 7:**
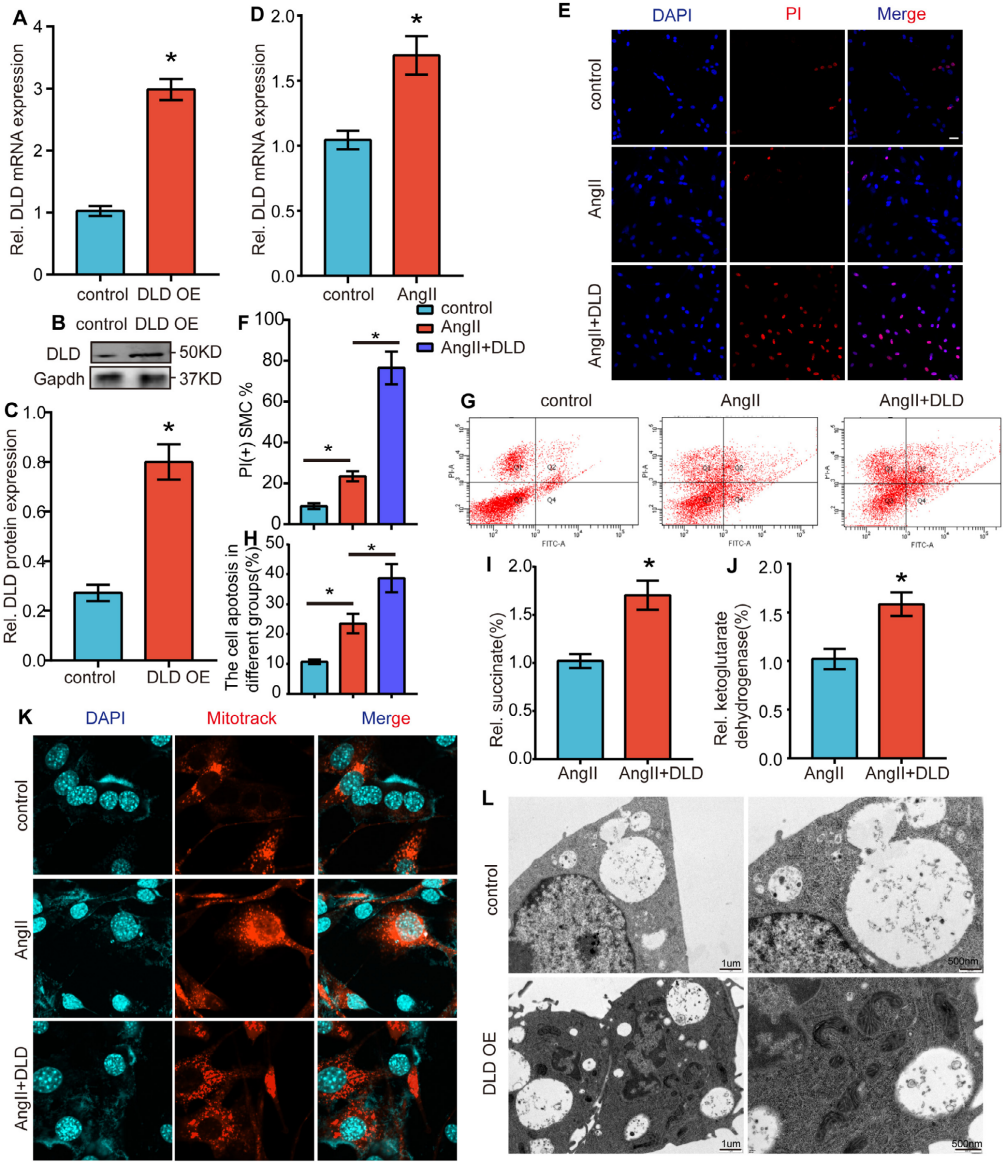
DLD regulates the necrosis, apoptosis and mitophagy of SMCs. (A) QRT‐PCR assay for detecting the expression level of DLD in VSMCs after transfected with DLD overexpressed plasmid. *p < 0.05 versus control; *n* = 5 per group. (B, C) Western blotting results and densitometric analysis of DLD expression level in control group and DLD overexpressed group. *n* = 4, *p < 0.05 versus control group. (D) QRT‐PCR assay for detecting the expression level of DLD in SMCs after AngII stimulation. (E, F) PI staining in SMCs after interference of DLD expression. The red dots indicate PI‐positive SMCs. *p < 0.05 versus PBS; *n* = 5 per group. (G, H) The results of the flow cytometry analysis and the relative expression in SMCs after transfection with AngII or AngII+DLD overexpressed plasmid. *n* = 4, *p < 0.05 versus scr‐RNA group. (I, J) Quantification of succinate and ketoglutarate dehydrogenase in SMCs stably transduced with DLD overexpressed plasmid or vector. The SMCs were also exposed to AngII. *p < 0.05 versus PBS; *n* = 5 per group. (K) Mitotrack staining of SMCs after transfection with AngII or AngII+DLD overexpressed plasmid. *n* = 4, *p < 0.05 versus scr‐RNA group. (L) Electron microscopy of the pyroptosis level of macrophages in control group and DLD overexpressed group.

To investigate the role of DLD in the physiological processes of HASMCs in vitro, a comprehensive experimental approach was undertaken. The overexpression of DLD resulted in a significant elevation in its protein levels, as evidenced by both polymerase chain reaction (PCR) (Figure 7A) and western blot analyses (Figure 7B,C). Additionally, PCR results indicated that treatment with angiotensin II (AngII) could lead to the upregulation of DLD in SMCs (Figure 7D). Subsequent examination via propidium iodide (PI) staining and flow cytometry disclosed that the overexpression of DLD markedly increased the rates of necrosis (Figure 7E,F) and apoptosis (Figure 7G,H) in VSMCs under standard stimulation conditions. Given the established involvement of DLD in the tricarboxylic acid (TCA) cycle, our study observed a notable increase in the concentrations of succinic acid and ketoglutarate dehydrogenase with DLD overexpression (Figure 7I,J). Moreover, it was observed that AngII induced mitophagy in SMCs, an effect that was mitigated by the overexpression of DLD (Figure 7K). Collectively, these observations imply a multifaceted role of DLD in cellular metabolism and the survival mechanisms of HASMCs, potentially mediated through its participation in the TCA cycle and regulation of mitophagy.

We further demonstrated the effect of DLD on macrophages and found that overexpression of DLD led to an increase in pyroptosis levels in these cells. This suggests that DLD may not only act on smooth muscle cells but could also have a similar effect on macrophages (Figure 7L).

### 3.6 Immune Infiltration Analysis Between AAA and Controls by CIBERSORT and ssGSEA Algorithm

**FIGURE 8:**
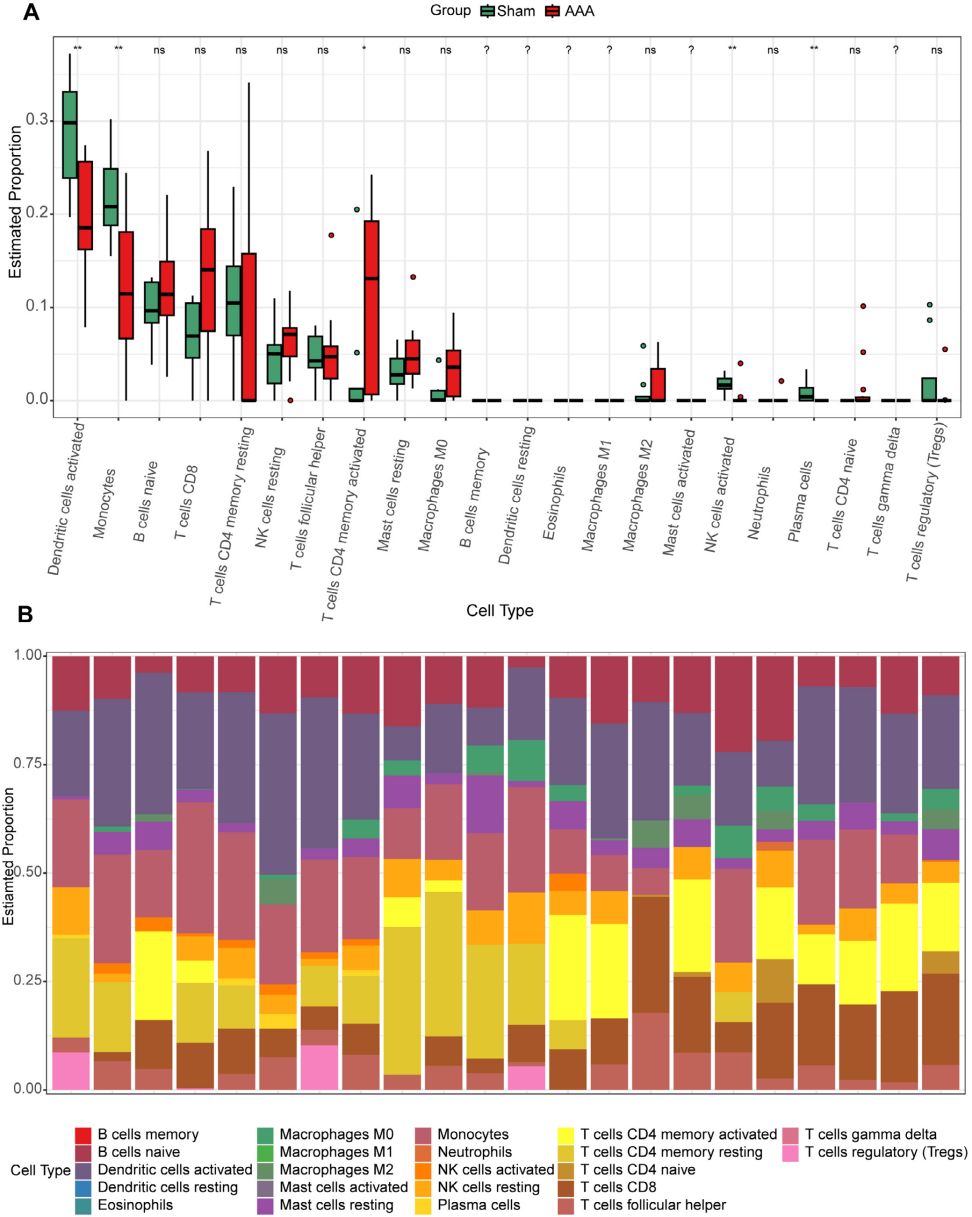
Immune infiltration analysis between AAA and controls by CIBERSORT algorithm. (A) Boxplots indicated the differences in immune infiltrating between AAA and control samples. *p < 0.05; **p < 0.01; ***p < 0.001. (B) The relative abundances of 22 infiltrated immune cells between AAA and control samples.

**FIGURE 9:**
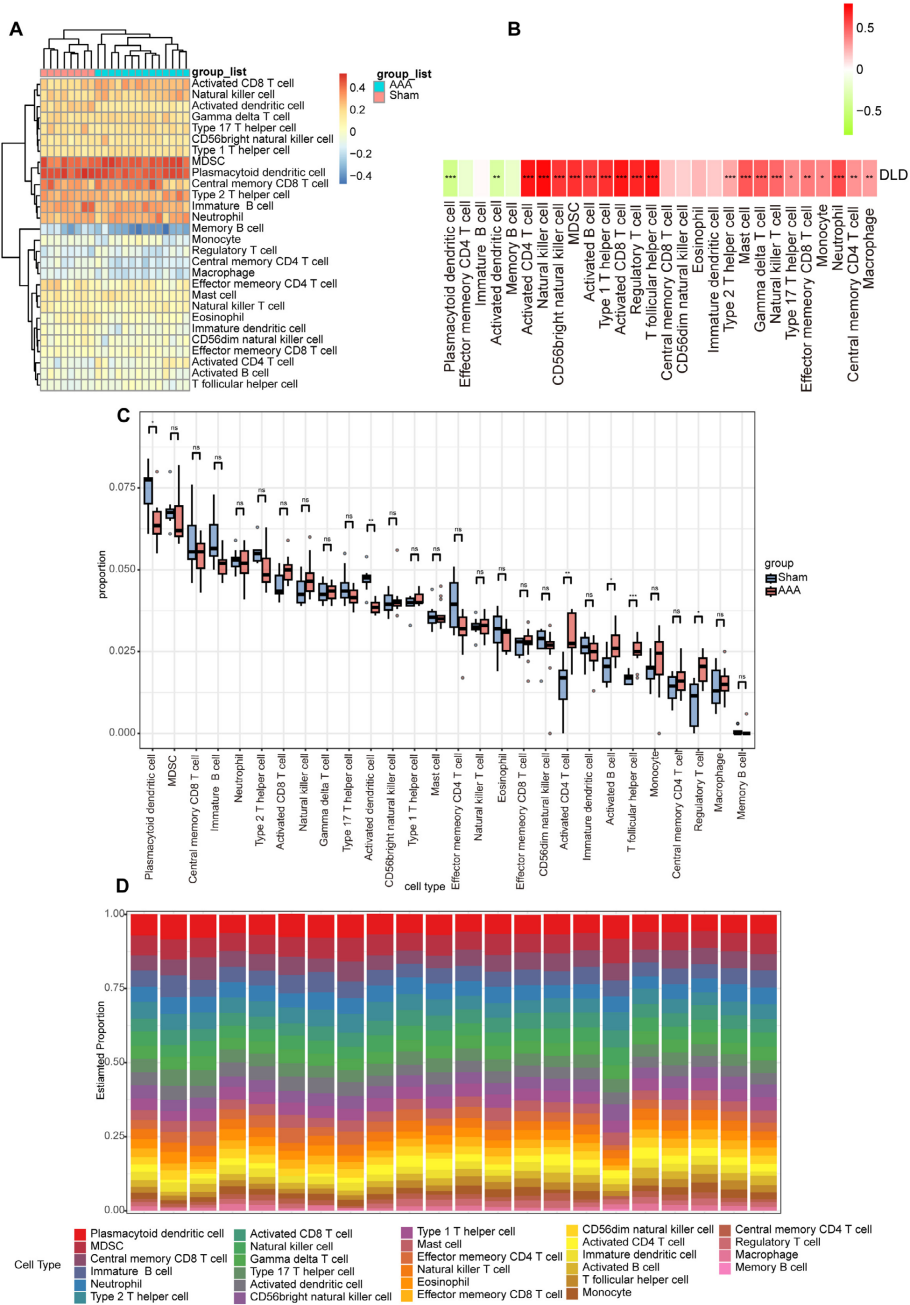
Immune infiltration analysis between AAA and controls by ssGSEA algorithm. (A) Heatmap of 28 immune cells between AAA and control samples by ssGSEA algorithm. (B) The correlation between 28 immune cells and DLD. Red and green colours represent positive and negative correlations respectively. *p*‐values are shown as: *p < 0.05; **p < 0.01; ***p < 0.001. (C) Boxplots indicated the differences in immune cells between AAA and control samples. *p < 0.05; **p < 0.01; ***p < 0.001. (D) The relative abundances of 28 infiltrated immune cells between AAA and control samples.

**FIGURE 10:**
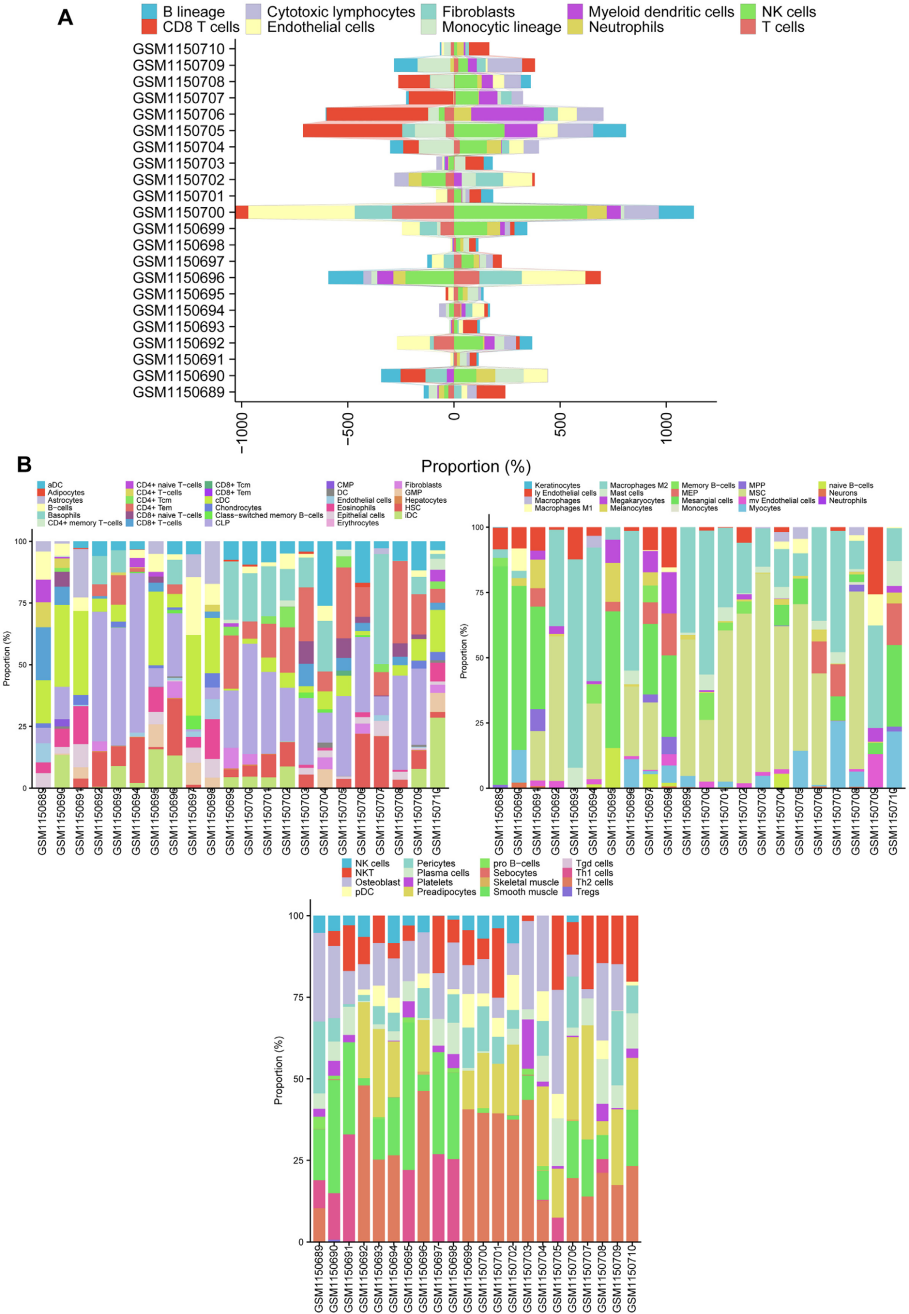
Immune infiltration analysis between AAA and controls by MCP‐counter and xCell algorithm. (A) The relative abundances of 10 infiltrated immune cells between AAA and control samples by MCP‐counter algorithm. (B) The relative abundances of 64 infiltrated immune cells between AAA and control samples by xCell algorithm.

We used the CIBERSORT and ssGSEA package (R software) to analyse immune cell infiltration in the samples, and 14 AAA samples and 8 normal aortic samples extracted from the GSE47472 dataset that met the standard for FRG expression were selected for this analysis (*p* < 0.05). We found significant differences in the infiltration ratio of activated dendritic cells, monocytes, activated CD4 memory T cells, activated NK cells and plasma cells between the normal aortic and AAA samples by CIBERSORT (Figure 8A). Specifically, we showed the proportion of immune cells in different individuals by CIBERSORT. The results showed that activated dendritic cells, monocytes and resting memory CD4 T cells accounted for the largest proportion of the 22 types of immune cells (Figure 8B). Heat map showing 28 types of immune cells between the five through ssGSEA in eight normal aortic samples and 14 AAA samples (Figure 9A). In the investigation of the correlation between DLD and various immune cell populations, the findings revealed a positive correlation between DLD and the following immune cell types: activated CD4 T cell, natural killer cell, CD56bright natural killer cell, myeloid‐derived suppressor cell (MDSC), activated B cell, type 1 T helper cell, activated CD8 T cell, regulatory T cell, T follicular helper cell, mast cell, natural killer T cell, gamma delta T cell, type 17 T helper cell, effector memory CD8 T cell, monocyte, neutrophil, central memory CD4 T cell and macrophage. Conversely, DLD exhibited a negative correlation with plasmacytoid dendritic cell (Figure 9B). We found significant differences in the infiltration ratio of plasmacytoid dendritic cell, activated dendritic cell, activated CD4 T cell, activated B cell T follicular helper cell and regulatory T cell between the normal aortic and AAA samples (Figure 9C). To provide a comprehensive visualisation of immune cell proportions across individuals, ssGSEA was employed, yielding a representative depiction (Figure 9D). In addition, we also incorporate additional analysis methods, such as MCP‐counter and xCell, for further analysis (Figure 10A,B). This systematic analysis enhances our understanding of immune cell dynamics in AAA pathology and underscores specific cellular interactions that may contribute to disease progression.

## 4 Discussion

In our current research, we initially identified DLD as a gene that is shared cuproptosis‐ with ferroptosis‐related genes in AAA. Subsequently, we confirmed an upregulation of DLD in both human and mouse AAA samples. Furthermore, our findings indicate that DLD plays a regulatory role in the necrosis, apoptosis and mitophagy of SMCs. Given these observations, DLD could potentially serve as a novel biomarker and a therapeutic target for AAA.

In our research, we identified that CRDEGs potentially play a role in the development of AAA. Through PPI and differential gene expression analyses, we discovered that FDX1, DLD, DLAT, LIAS, MTF1 and PDHA1 exhibited differential expression between AAA and control groups. Notably, we observed significant correlations among these CRDEGs, particularly MTF1, which showed a negative correlation with FDX1 and positive correlations with both DLD and LIAS. These correlations imply that genes associated with cuproptosis might be concurrently involved in AAA pathogenesis. Further analysis using GO and KEGG pathways indicated that these CRDEGs are predominantly engaged in ‘Glycolysis/Gluconeogenesis’, ‘Citrate cycle (TCA cycle)’ and ‘Pyruvate metabolism’, suggesting that they may influence AAA development through the modulation of glucose metabolic processes. Previous research has highlighted the importance of cuproptosis‐related genes in various pathological conditions. For instance, the occurrence of cuproptosis in Crohn's disease is associated with immune cell infiltration and metabolic alterations, suggesting that cuproptosis could contribute to the progression of Crohn's disease by triggering immunological responses and metabolic disarray [14]. This information provides a basis for further investigation into the role of cuproptosis in AAA and other diseases, potentially opening new avenues for therapeutic intervention.

In our study, we identified DLD as a DEG that is associated with both cuproptosis and ferroptosis in AAA. In this study, we overlapped DEG with ferroptosis‐related genes in GeneCard and screened out that five FRDEGs (DLD, NEDD4L, PVT1, SCL38A1 and ZFP36) might be involved in the AAA formation, which suggesting that these FRDEGs might be involved in the formation of AAA by regulating ferroptosis. Previous studies showed that FRDEGs (GPX4, IL‐6, PRXD1 and SCD) might regulate the pathological process of AAA [9], which further supports that ferroptosis plays an important role in AAA. We found correlations between CRDEGs and FRDEGs, which suggested that there are molecular cross‐talk and interaction between cuproptosis and ferroptosis. In addition, previous studies have identified similarities between ferroptosis and cuproptosis, both being triggered by reactive oxygen species (ROS), which further supports the current findings. By overlapping CRDEGs with FRDEGs, one gene, DLD, was identified as an overlapping cuproptosis‐ and ferroptosis‐related DEG. Further investigation revealed that DLD was highly expressed in human and mouse AAA samples, suggesting its potential as a biomarker for predicting AAA occurrence.

Our research findings suggest that DLD exerts a regulatory influence on the necrotic, apoptotic and mitophagy in SMCs. The overexpression of DLD was correlated with an increased quantity of PI‐stained SMCs, as well as a heightened count of apoptotic cells, a result that is congruent with previous research indicating DLD's role in promoting PI staining of cells [21]. Studies have established a nexus between ferroptosis and cuproptosis with mitochondrial functionality [22]. Key to mitochondrial function are the enzymes succinic acid and ketoglutarate dehydrogenase, which participate in the TCA cycle [23]. Our investigation revealed that the overexpression of DLD augments the activity of these enzymes, a finding that aligns with existing literature. More intriguingly, our research has linked DLD to the process of mitophagy with increased DLD expression attenuating the incidence of this autophagic event. Previous investigations have demonstrated a positive correlation between cuproptosis‐related genes and the NLRP3 inflammasome in the context of AAA [24], suggesting that the inhibition of mitophagy can lead to the activation of the NLRP3 inflammasome. This correlation implies that genes associated with cuproptosis may play a role in the aetiology of mitophagy, a notion that is reinforced by our findings. Collectively, these observations point to a complex interplay between DLD, mitochondrial function and cell death pathways in SMCs, which could have significant implications for the understanding and treatment of AAA.

The present study acknowledges several limitations that warrant consideration. Firstly, our study has not yet included in vivo experimental validation to determine whether DLD could indeed promote the development of AAA in a murine model. Secondly, the precise molecular mechanisms through which DLD might induce cuproptosis, and its subsequent implications in AAA pathogenesis, remain to be elucidated.

To summarise, our study initially identified DLD as a gene that is associated with both cuproptosis and ferroptosis, and is differentially expressed in the context of AAA. Subsequent analyses confirmed the upregulation of DLD in AAA samples from both human and murine sources. Our research further suggests that DLD exerts regulatory effects on the necrosis, apoptotic and mitophagy in SMCs. Considering these findings, DLD may emerge as a promising biomarker and a potential therapeutic target for AAA, offering new avenues for clinical intervention and patient management.

## Author Contributions

**Xingwei Hu:** conceptualization (equal), methodology (equal), writing – original draft (equal). **Lu Hu:** conceptualization (equal), methodology (equal), writing – original draft (equal). **Xiaoyun Si:** formal analysis (equal), investigation (equal). **Qian Feng:** formal analysis (equal), investigation (equal). **Yi Ma:** supervision (equal). **Zhijiang Liu:** supervision (equal). **Xiang He:** conceptualization (equal), writing – review and editing (equal). **Bei Shi:** conceptualization (equal), funding acquisition (lead), supervision (equal), writing – review and editing (equal).

## Ethics Statement

Aneurysm samples and adjacent normal aortic tissues were collected from AAA patients in NanFang Hospital who underwent AAA resection surgery. All protocols involving human specimens were approved by NanFang Hospital [19] (ethical approval number: NFEC‐2019‐086).

## Conflicts of Interest

The authors declare no conflicts of interest.

## Supporting information


**Table S1.** Antibodies for immunohistochemistry analysis.
**Table S2**. Antibodies for western blots.

## Data Availability

Data will be made available on request.

## References

[1] W. Lu , Y. Zhou , S. Zeng , et al., “Loss of FoxO3a Prevents Aortic Aneurysm Formation Through Maintenance of VSMC Homeostasis,” Cell Death & Disease 12, no. 4 (2021): 378. 33828087 10.1038/s41419-021-03659-y PMC8027644

[2] M. P. Tigga and G. G. Gowda , “A Sinister Gut Feeling,” Journal of Mid‐Life Health 12, no. 3 (2021): 247–249. 34759710 10.4103/jmh.JMH_10_19 PMC8569457

[3] K. Yoshimura , N. Morikage , S. Nishino‐Fujimoto , A. Furutani , B. Shirasawa , and K. Hamano , “Current Status and Perspectives on Pharmacologic Therapy for Abdominal Aortic Aneurysm,” Current Drug Targets 19, no. 11 (2018): 1265–1275. 29284386 10.2174/1389450119666171227223331 PMC6182934

[4] F. A. Lederle , R. L. Kane , R. MacDonald , and T. J. Wilt , “Systematic Review: Repair of Unruptured Abdominal Aortic Aneurysm,” Annals of Internal Medicine 146, no. 10 (2007): 735–741. 17502634 10.7326/0003-4819-146-10-200705150-00007

[5] G. Accarino , A. N. Giordano , M. Falcone , et al., “Abdominal Aortic Aneurysm: Natural History, Pathophysiology and Translational Perspectives,” Translational Medicine @ UniSa 24, no. 2 (2022): 30–40. 37476203 10.37825/2239-9747.1037 PMC10354862

[6] H. Y. Tian , B. Y. Huang , H. F. Nie , et al., “The Interplay Between Mitochondrial Dysfunction and Ferroptosis During Ischemia‐Associated Central Nervous System Diseases,” Brain Sciences 13, no. 10 (2023): 1367. 37891735 10.3390/brainsci13101367 PMC10605666

[7] F. Zhang , K. Li , W. Zhang , et al., “Ganglioside GM3 Protects Against Abdominal Aortic Aneurysm by Suppressing Ferroptosis,” Circulation 149, no. 11 (2024): 843–859. 38018467 10.1161/CIRCULATIONAHA.123.066110

[8] H. Wu , L. Chen , K. Lu , et al., “HMGB2 Deficiency Mitigates Abdominal Aortic Aneurysm by Suppressing Ang‐II‐Caused Ferroptosis and Inflammation via NF‐Kappabeta Pathway,” Mediators of Inflammation 2023 (2023): 2157355. 38148870 10.1155/2023/2157355 PMC10751175

[9] K. Wang , Y. Song , H. Li , J. Song , and S. Wang , “Identification of Differentially Expressed Ferroptosis‐Related Genes in Abdominal Aortic Aneurysm: Bioinformatics Analysis,” Frontiers in Cardiovascular Medicine 9 (2022): 991613. 36247434 10.3389/fcvm.2022.991613 PMC9558826

[10] J. Ren , Y. Lv , L. Wu , et al., “Key Ferroptosis‐Related Genes in Abdominal Aortic Aneurysm Formation and Rupture as Determined by Combining Bioinformatics Techniques,” Frontiers in Cardiovascular Medicine 9 (2022): 875434. 36017103 10.3389/fcvm.2022.875434 PMC9395677

[11] J. Wang , L. Z. Luo , D. M. Liang , et al., “Progress in the Research of Cuproptosis and Possible Targets for Cancer Therapy,” World Journal of Clinical Oncology 14, no. 9 (2023): 324–334. 37771632 10.5306/wjco.v14.i9.324 PMC10523190

[12] M. Yang , Y. Wang , L. He , X. Shi , and S. Huang , “Comprehensive Bioinformatics Analysis Reveals the Role of Cuproptosis‐Related Gene Ube2d3 in Myocardial Infarction,” Frontiers in Immunology 15 (2024): 1353111. 38440726 10.3389/fimmu.2024.1353111 PMC10909922

[13] Z. Liu , L. Wang , Q. Xing , et al., “Identification of GLS as a Cuproptosis‐Related Diagnosis Gene in Acute Myocardial Infarction,” Frontiers in Cardiovascular Medicine 9 (2022): 1016081. 36440046 10.3389/fcvm.2022.1016081 PMC9691691

[14] Y. Yuan , M. Fu , N. Li , and M. Ye , “Identification of Immune Infiltration and Cuproptosis‐Related Subgroups in Crohn's Disease,” Frontiers in Immunology 13 (2022): 1074271. 36466876 10.3389/fimmu.2022.1074271 PMC9713932

[15] J. Li , F. Cao , H. L. Yin , et al., “Ferroptosis: Past, Present and Future,” Cell Death & Disease 11, no. 2 (2020): 88. 32015325 10.1038/s41419-020-2298-2 PMC6997353

[16] B. Guo , F. Yang , L. Zhang , et al., “Cuproptosis Induced by ROS Responsive Nanoparticles With Elesclomol and Copper Combined With alphaPD‐L1 for Enhanced Cancer Immunotherapy,” Advanced Materials 35, no. 22 (2023): e2212267. 36916030 10.1002/adma.202212267

[17] C. Zhao , Z. Zhang , and T. Jing , “A Novel Signature of Combing Cuproptosis‐ With Ferroptosis‐Related Genes for Prediction of Prognosis, Immunologic Therapy Responses and Drug Sensitivity in Hepatocellular Carcinoma,” Frontiers in Oncology 12 (2022): 1000993. 36249031 10.3389/fonc.2022.1000993 PMC9562991

[18] X. He , S. Wang , M. Li , et al., “Long Noncoding RNA GAS5 Induces Abdominal Aortic Aneurysm Formation by Promoting Smooth Muscle Apoptosis,” Theranostics 9, no. 19 (2019): 5558–5576. 31534503 10.7150/thno.34463 PMC6735383

[19] X. He , X. Li , Y. Han , et al., “CircRNA Chordc1 Protects Mice From Abdominal Aortic Aneurysm by Contributing to the Phenotype and Growth of Vascular Smooth Muscle Cells,” Molecular Therapy ‐ Nucleic Acids 27 (2022): 81–98. 34938608 10.1016/j.omtn.2021.11.005 PMC8649900

[20] L. Zhong , X. He , H. Song , et al., “METTL3 Induces AAA Development and Progression by Modulating N6‐Methyladenosine‐Dependent Primary miR34a Processing,” Molecular Therapy ‐ Nucleic Acids 21 (2020): 394–411. 32650237 10.1016/j.omtn.2020.06.005 PMC7347714

[21] D. Shin , J. Lee , J. H. You , D. Kim , and J. L. Roh , “Dihydrolipoamide Dehydrogenase Regulates Cystine Deprivation‐Induced Ferroptosis in Head and Neck Cancer,” Redox Biology 30 (2020): 101418. 31931284 10.1016/j.redox.2019.101418 PMC6957841

[22] J. Wang , J. Li , J. Liu , et al., “Interplay of Ferroptosis and Cuproptosis in Cancer: Dissecting Metal‐Driven Mechanisms for Therapeutic Potentials,” Cancers (Basel) 16, no. 3 (2024): 512. 38339263 10.3390/cancers16030512 PMC10854932

[23] G. Morris , M. Maes , M. Berk , and B. K. Puri , “Myalgic Encephalomyelitis or Chronic Fatigue Syndrome: How Could the Illness Develop?,” Metabolic Brain Disease 34, no. 2 (2019): 385–415. 30758706 10.1007/s11011-019-0388-6 PMC6428797

[24] J. Hu , S. Xue , Z. Xu , et al., “Identification of Core Cuprotosis‐Correlated Biomarkers in Abdominal Aortic Aneurysm Immune Microenvironment Based on Bioinformatics,” Frontiers in Immunology 14 (2023): 1138126. 37138870 10.3389/fimmu.2023.1138126 PMC10150024

