# Supplementary material for: Comprehensive Bioinformatics Analysis Reveals the Role of Shared Cuproptosis‐ and Ferroptosis‐Related DEG DLD in Abdominal Aortic Aneurysm
Source: J Cell Mol Med. 2025 Feb 6;29(3):e70399. doi: 10.1111/jcmm.70399 (PMC11799913; doi:10.1111/jcmm.70399)
Supplement: Supplementary file 1 — Table S1. Antibodies for immunohistochemistry analysis. Table S2. Antibodies for western blots. [file JCMM-29-e70399-s001.docx]

**Table S1. Antibodies for immunohistochemistry analysis**

| name | Vendor or Source | Catalog # | dilution factors | relevant references supporting antibody validation |  |
| --- | --- | --- | --- | --- | --- |
| Anti-αSMA | abcam | ab5694 | BSA  1:1000 | PMID: 37928264 |  |
| Anti-DLD | GeneTex | GTX101245 | BSA  1:1000 | PMID: 31931284 |  |
| **Table S2. Antibodies for western blots** | | | | | |
| name | Vendor or Source | Catalog # | dilution factors | relevant references supporting antibody validation | |
| Anti-DLD | GeneTex | GTX101245 | BSA  1:1000 | PMID: 31931284 | |
